# The long-term outcome and risk factors of histologic discrepancy between forceps biopsies and endoscopic resections in early gastric cancer: An observational study

**DOI:** 10.1097/MD.0000000000038451

**Published:** 2024-06-07

**Authors:** Min-Kyung Yeo, Jae Ho Park, Sun Hyung Kang, Hee Seok Moon, Jae Kyu Sung, Hyun Yong Jeong, Ju Seok Kim

**Affiliations:** a Department of Pathology, Chungnam National University College of Medicine, Daejeon, Korea; b Department of Internal Medicine, Chungnam National University College of Medicine, Daejeon, Korea

**Keywords:** early gastric cancer, endoscopic forceps biopsy, endoscopic resection

## Abstract

Although endoscopic forceps biopsy is the gold standard for early gastric cancer (EGC) diagnosis, the method can cause endoscopic resection of specimens and histological discrepancies. This study aims to examine the risk factors for histological discrepancies in EGC and long-term clinical outcomes.

This retrospective study included patients diagnosed with differentiated-type EGC using forceps biopsy. Patients without histological discrepancies and with undifferentiated types in endoscopic resection histology were categorized into the concordant and discordant groups, respectively. Clinical characteristics and long-term outcomes related to histological discrepancies were analyzed.

A total of 957 lesions from 936 patients were enrolled. An overall discrepancy rate of 8.7% was confirmed, with an undifferentiated-type discrepancy of 5.5%. The discordant group showed a higher tendency for lesions to be located in the upper third region, to have whitish discoloration, and to undergo a greater number of biopsies compared with the concordant group. Multivariate analysis confirmed that lesion location in the upper third region (odds ratio [OR]: 2.125; 95% confidence interval [CI]: 1.032–5.277; *P* = .041) and whitish surface discoloration (OR: 13.615; 95% CI: 6.028–28.728; *P* = .001) were significantly correlated with histologic discrepancy. Compared with the concordant group, the discordant group had a lower curative resection rate, but no differences were observed in complications, local recurrence, or survival rates.

Upper third location and whitish discoloration were risk factors for the histologic discrepancy between differentiated and undifferentiated types in patients with EGC. For curative resections performed in patients with EGC and histologic discrepancies and without additional treatment, careful follow-up is possible.

## 1. Introduction

Currently, the methods of endoscopic submucosal dissection (ESD) and endoscopic mucosal resection (EMR) result in satisfactory treatment results. Furthermore, since these methods are associated with a low rate of complications, they are used as effective standard treatment methods in many countries.^[1,2]^ For an absolute indication of the endoscopic resection, elevated lesions in differentiated-type adenocarcinoma that do not accompany ulcers should be less than or equal to 2 cm, whereas, in the case of depressed lesions, ulcers less than or equal to 1 cm are observed. Since the absolute indication is highly strict and can require unnecessary surgeries, there are cases where the indication is enlarged to conduct endoscopic resections.^[3]^ Recently, there have been attempts to conduct endoscopic resection even for undifferentiated early gastric cancers that are <2 cm. However, this method is controversial due to the claims that, in this method, there is a higher probability of local recurrence and lymph node metastasis in comparison to that in the absolute indication group.^[4,5]^ The appropriate treatment method can differ depending upon the histological categorization, and for all EGC lesions that are under consideration for endoscopic resections, forceps biopsies are usually conducted before the surgery to confirm the specific histological differentiation.

However, forceps biopsies are limited because of the partial analysis of a subset of lesions, generating the possibility of sampling error and discrepancies in the final histological results after endoscopic resection.^[6–8]^ Previous studies have shown that 1.5% to 7.0% of patients diagnosed with differentiated EGC in the forceps biopsy have a histological discrepancy and are finally confirmed to have undifferentiated EGC.^[9,10]^ However, in such conditions, exact guidelines for determining the requirement of additional surgery or continuing without additional treatment are lacking. Furthermore, information on the risk factors for histological discrepancies in patients with EGC is not available. This study aimed to identify the risk factors for histological discrepancies in patients with EGC and their long-term clinical outcomes.

## 2. Methods

### 2.1. Patients

A retrospective chart analysis was conducted on patients who underwent endoscopic resection of differentiated-type EGC using forceps biopsies between January 2015 and December 2021 at Chungnam National University Hospital (Daejeon, Korea). This data included patients that had initial forceps biopsies performed at other hospitals and were later transferred to our hospital, and the analysis was conducted on patients that had a follow-up period of at least 1 year. Patients beyond absolute indications, especially those with large lesions or suspected submucosal invasion, were excluded from the analysis. Patients who were not diagnosed with EGC after endoscopic resection (non-neoplastic or adenoma) were also excluded from the analysis.

According to the World Health Organization classifications, patients are categorized into differentiated (papillary, well, and moderately differentiated) and undifferentiated (poorly differentiated adenocarcinoma or signet ring cell carcinoma) types. The macroscopic type of EGC was categorized according to the macroscopic classification of the Japanese Gastric Cancer Association.^[11]^ When the final histological results of the endoscopic resection and forceps biopsies matched, the patients were categorized into the concordant group, and when the patient was diagnosed as a differentiated type in the forceps biopsy, but as an undifferentiated type after the endoscopic resection, the patients were categorized into the discordant group. The clinical and histological characteristics and short- and long-term therapeutic outcomes after endoscopic resection were comparatively analyzed in both groups. This study was a retrospective study that analyzed only the patient’s medical records, and approval from the Institutional Review Board was not necessary.

### 2.2. Endoscopic resection

All patients underwent endoscopic resection (EMR or ESD) performed by an expert gastrointestinal (GI) endoscopist. Chromoendoscopy with the indigo carmine solution was used to confirm the boundaries of the lesions; when necessary, narrow-band imaging was also conducted. Endoscopic EMRs and endoscopic ESDs were performed using standard methods. For ESDs, an insulation-tipped diathermy (IT) knife or IT knife-2 (Olympus Medical, Tokyo, Japan) was used, while high-frequency generators (ICC200 or VIO 300D; ERBE Elekromedizin, Tubingen, Germany) were used for endoscopic resections. After the endoscopic resections were performed, all patients were hospitalized and underwent complete blood cell count tests, and abdominal radiography was performed to examine any complications in the procedures.

### 2.3. Pathologic diagnosis

Tissue samples were obtained from all lesions suspected to be EGC from the initial endoscopy using standard biopsy forceps. For patients transferred after undergoing biopsies at other hospitals, additional biopsies were either not performed or performed at a minimum because conducting another biopsy could cause ulcers or fibrosis that might interfere with endoscopic resection. Most external hospital tissue examination slides were reviewed again at our hospital. Tissue samples from the endoscopic resection were spread and fixed on a polystyrene plate using pins and fixed in 10% formalin. After sectioning the samples every 2 mm, experienced GI pathologists examined the samples under a microscope. When more than 1 type of cell tissue was included, the histological types were categorized using the cells that comprised more tissue.

### 2.4. Clinical outcome

*En bloc* resection was defined as the surgical removal of 1 piece of tissue that had undergone ESD or EMR. There were no lymphovascular invasions in the curative resection, and even when *En bloc* resection was performed on the removed tissue with a lateral margin of more than 2 mm and a basal margin of more than 0.5 mm, or even when the tissue was removed piecemeal if the tissue pieces could be confirmed and examined through complete reconstruction, these tissue samples were included in the curative resection. Patients were checked for bleeding and perforation complications after the procedure. Bleeding was defined as the presence of melena or hematemesis, additional endoscopic hemostatic procedures were necessary, or when hemoglobin levels dropped to below 2 g/dl; perforation was defined as cases discovered during the endoscopic resection or through the radiograph after the procedure had been completed. Local recurrence was defined as cancer recurrence 12 months after undergoing endoscopic resection; cases in which the patient died during the follow-up period were categorized according to the cause of death.

### 2.5. Statistical analysis

Continuous variables are presented as means and standard deviations (SD), and categorical variables are presented as frequencies and percentages. By dividing the patients into 2 groups depending on the histologic discrepancy, patient characteristics and clinical outcomes were comparatively analyzed using the Chi-square test or Fisher exact test. To derive the risk factors for the histologic discrepancy, the odds ratio (OR) and the confidence interval (CI) were indicated through logistic regression analysis by including multivariate models when the *P*-values ≤ 0.1 in the univariate analysis. All analyses were performed using SPSS software (version 18.0 (SPSS Inc., Chicago, IL, USA), and a *P*-value (two-sided) of < 0.05 was considered significant.

## 3. Results

### 3.1. Patient characteristics

**Figure 1.**
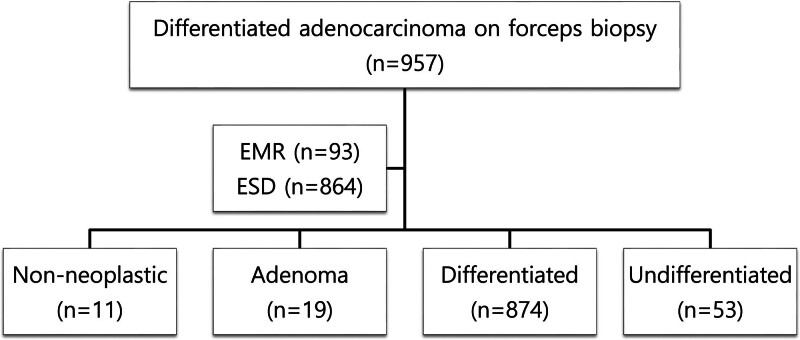
Flow chart of total patients.

A total of 957 lesions were enrolled in 938 patients who had been diagnosed with differentiated-type EGC by forceps biopsy, including 673 (71.7%) patients who were diagnosed and transferred from other hospitals. Of the 673 patients transferred to our hospital who underwent endoscopic resections, 389 (57.8%) underwent endoscopic resections based on a review of pathological slides from an external hospital without conducting an additional tissue examination. The final pathological results from endoscopic resections were confirmed as non-neoplastic (n = 11), adenoma (n = 19), differentiated type (n = 874), and undifferentiated type (n = 53) (Fig. 1). The results of this study confirmed that there were 874 and 53 patients in the concordant and discordant groups, respectively.

The overall discrepancy rate that included the non-neoplastic and adenoma groups was 8.7% (83/957); when only the undifferentiated type was examined, the rate was 5.5% (53/957). There were 72.5% (n = 693) males in the entire patient group, and the mean age (SD) was 64.2 (8.6) years (Table 1). Most lesions were confirmed to be in the lower third of the stomach (52.5%), and when categorized according to morphological characteristics, the macroscopic depressed type (69.8%) was the most common.

**Table 1:** Baseline patient characteristics

Characteristics, No. (%)	Total patients (n = 957)
Age, years (mean, SD)	64.2 (8.6)
Gender (male)	693 (72.5)
Location	
Upper third	117 (12.3)
Middle third	338 (35.2)
Lower third	502 (52.5)
Macroscopic type	
Elevated	131 (13.7)
Flat	157 (16.5)
Depressed	669 (69.8)
Endoscopic resection method	
EMR	93 (9.7)
ESD	864 (90.3)
Lesion diameter, mm (mean, SD)	14.3 (3.8)
Number of biopsy (mean, SD)	2.1 (0.8)
*H pylori* infection	409 (42.8)

EMR *=* endoscopic mucosal resection, ESD *=* endoscopic submucosal dissection, *H pylori = Helicobacter pylori*, SD *=* standard deviation

### 3.2. Concordant and discordant group

73.1% of the concordant group was males and the mean age (SD) was 63.8 (9.2) years old, while 71.9% of the discordant group was males and the mean age (SD) was 65.1 (11.4) years old, confirmed that there was no statistically significant difference between the 2 groups (Table 2). The macroscopic type (*P* = .635), endoscopic resection method (*P* = .332), lesion diameter (*P* = .280), presence of erosion (*P* = .888), and presence of *Helicobacter pylori* infection (*P* = .929) were also not significantly different between the 2 groups. However, when compared to the concordant group, it was confirmed that there was a statistically significant difference for the discordant group for the location of the lesion in the upper third of the stomach (22.6% vs 11.8%, *P* = .039), more of a whitish discoloration than erythema for the color tone change at the surface (71.7% vs 14.0%, *P* = .000), and there was also a tendency for a higher number of forceps biopsies (*P* = .041).

**Table 2:** Comparison of baseline characteristics between concordant and discordant group after endoscopic resection

Characteristics No. (%)	Concordant (n = 874)	Discordant (n = 53)	*P* value
Age, years (mean, SD)	63.8 (9.2)	65.1 (11.4)	0.375
Gender (male)	639 (73.1)	10 (71.9)	0.261
Location			0.039
Upper third	103 (11.8)	12 (22.6)	
Middle third	312 (35.7)	18 (33.9)	
Lower third	459 (52.5)	23 (43.5)	
Macroscopic type			0.635
Elevated	118 (13.5)	6 (11.3)	
Flat	158 (18.1)	13 (24.5)	
Depressed	598 (68.4)	34 (64.2)	
Endoscopic resection method			0.332
EMR	73 (8.4)	7 (13.2)	
ESD	801 (91.6)	46 (86.8)	
Lesion diameter, mm (mean, SD)	13.9 (3.9)	15.2 (4.5)	0.280
Surface color			0.000
Normal or erythema	752 (86.0)	15 (28.3)	
Whitish discoloration	122 (14.0)	38 (71.7)	
Presence of erosion	53 (6.1)	4 (7.5)	0.888
Number of biopsy (mean, SD)	2.0 (0.9)	2.3 (1.2)	0.041
*H pylori* infection	377 (43.1)	22 (41.5)	0.929

EMR *=* Endoscopic mucosal resection, ESD *=* Endoscopic submucosal dissection, *H pylori =* Helicobacter pylori, SD *=* Standard deviation

Multivariate analysis was conducted by adjusting for location, surface color, and the number of biopsies, which were confirmed to be statistically significant in the univariate analysis (Table 3). The location of the lesions was confirmed to be more frequent in the upper third of the stomach (OR: 2.125; 95% CI: 1.032–5.277, *P* = .041) for the discordant group when compared to that of the concordant group, and the whitish discoloration on the surface (OR: 13.615; 95% CI: 6.028–28.728, *P* = .001) was confirmed to be statistically significantly higher. However, no differences were confirmed for the middle third location (OR: 1.081; 95% CI: 0.524–2.961, *P* = .871) or the number of forceps biopsies (OR: 1.153; 95% CI: 0.691–3.173, *P* = .522) between the 2 groups.

**Table 3:** Multivariate analysis of factors associated with the histologic discrepancy between forceps biopsy and endoscopic resection

Variables	OR (95% CI)	*P* value*
Location		
Lower third	1	
Middle third	1.081 (0.524–2.961)	0.871
Upper third	2.125 (1.032–5.277)	0.041
Surface color		
Normal or erythema	1	
Whitish discoloration	13.615 (6.028–28.728)	0.001
Number of biopsy	1.153 (0.691–3.173)	0.522

CI = confidence interval, OR *=* odds ratio.

* Adjusted for location, surface color and number of biopsy.

### 3.3. Short and long-term outcomes

Short- and long-term clinical outcomes were confirmed after endoscopic resection (Table 4). The *En bloc* resection rates were 92.1% (805/874) and 89.4% (47/53) in the concordant and discordant groups, respectively, with no significant difference between the 2 groups (*P* = .529). The resection margin invasion rates for the 2 groups were 2.0% (18/874) and 5.7% (n = 3), respectively, confirming that there was no significant difference between the groups (*P* = .240). Comparing the complications that occurred after endoscopic resections, bleeding (4.5 %) and perforation (0.6 %) occurred in the concordant group, and bleeding (7.5 %) and perforation (0.0 %) occurred in the discordant group, confirming that there was no statistically significant difference between the 2 groups (*P* = .658).

**Table 4:** Clinical outcome of the endoscopic resection, according to the histological discrepancy

Characteristics, No. (%)	Concordant (n = 874)	Discordant (n = 53)	*P* value
*En bloc* resection	805 (92.1)	47 (89.4)	0.529
Curative resection	845 (96.7)	46 (86.8)	0.002
Margin positive	18 (2.0)	3 (5.7)	0.240
Lateral	11 (1.3)	2 (3.8)	
Basal	5 (0.5)	1 (1.9)	
Both	2 (0.2)	0 (0.0)	
Complication	45 (5.1)	4 (7.5)	0.658
Bleeding	39 (4.5)	4 (7.5)	
Perforation	6 (0.6)	0 (0.0)	
Local recurrence	3 (0.3)	1 (1.9)	0.558
Death	8 (0.9)	1 (1.9)	1.000
Cancer related	5 (0.5)	1 (1.9)	
Other cause	3 (0.4)	0 (0.0)	

During the mean (SD) 37.82 (11.2-) month follow-up period, local recurrence was observed in 4 patients. Three of these patients were from the concordant group, and 1 patient was from the discordant group, confirming that there was no statistically significant difference between the 2 groups (*P* = .558). The 5-year disease-specific and recurrence-free rates were 98.7% and 95.5% for the concordant and discordant groups, respectively. Two patients with local recurrence underwent additional endoscopic resection, whereas the other 2 patients underwent surgical resection. During the same follow-up, 9 patients died, of which 6 patients died from cancer-related causes and 3 from other causes. Of these 9 patients, 8 were from the concordant group and 1 was from the discordant group, confirming that there was no statistically significant difference between the 2 groups (*P* = 1.000). The 5-year overall survival rates were 95.1% and 93.7% in the concordant and discordant groups, respectively.

## 4. Discussion

In the present study, 5.5% of all cases showed histologic discrepancies, which is consistent with previous reports.^[9,10]^ This was due to differentiated-type diagnosis made in the forceps biopsies initially, but later diagnosed as undifferentiated type after the endoscopic resection. Histologic discrepancies can be caused by mixing of multiple histologic types in the lesion. Since forceps biopsies only extract a partial subset of the lesions, they may yield pathological results different from that of the entire tissue, also causing a difference in the sampling error.^[6–8]^ Magnifying narrow-band imaging endoscopy is a type of method introduced to reduce histologic discrepancy. Although this method is shown to be partially helpful in reducing histologic discrepancy, there is controversy regarding its clinical adoption.^[12,13]^

Although different factors have been associated with histologic discrepancy in many studies, most of these studies are related to gastric adenoma, and studies related to gastric cancer are lacking.^[9,14]^ According to a recent study, mid-third location of the stomach (OR: 5.34; 95% CI: 1.59–19.13; *P* = .012) and easy friability (OR: 29.26; 95% CI: 2.30–999.9; *P* = .009) were confirmed as the risk factor for histologic discrepancy.^[15]^ The multivariate analysis results in the present study confirmed that lesions located in the upper third of the stomach and the surface whitish discoloration were significant factors related to the histologic discrepancy. During endoscopy, lesions located in the upper third of the stomach with a discoloration closer to white than red, the possibility of histologic discrepancy must be considered. However, the research on histological discrepancies in EGC is not extensively studied, and hence further research is required.

This study confirmed that the number of forceps biopsies was not related to the histologic discrepancy. Although the number of forceps biopsies tended to be higher in the discordant group than in the concordant group, no significant differences were confirmed. In previous studies examining histological discrepancies in gastric cancer, the number of biopsies was confirmed to be unrelated to histological discrepancies (OR: 1.118; 95% CI: 0.977–1.444; *P* = .085).^[16]^ When there is suspicion of gastric cancer from the results of a gastroscopy, it is general practice to recommend 4–7 pieces of forceps biopsy.^[17,18]^ If the endoscopist determines that the lesion is an indication of the endoscopic resection from the results of the endoscopy and recommends an endoscopic resection, due to the possibility of ulceration or fibrosis after conducting a forceps biopsy, there is a tendency to avoid forceps biopsies. Therefore, the number of confirmed forceps biopsies in the present study was low. When considering the possibility of ulceration or fibrosis that can occur after a forceps biopsy, accurate targeted biopsies are considered to be more important than conducting a large number of forceps biopsies.

Few studies have examined the effect of histological discrepancies on clinical outcomes after endoscopic resections. A previous study compared the therapeutic outcomes of patients with histologic discrepancy from a differentiated type to an undifferentiated type who underwent endoscopic resections, and confirmed that the curative resection rate (*P* < .001) was low in the discordant group, while additional operations after performing endoscopic resections were significantly higher (*P* < .001).^[15]^ However, the authors of this research explained that this difference was caused because there were higher submucosal invasion and lymph-vascular invasion for the discordant group and not due to effects from the histologic difference. While no differences were confirmed in this study between the 2 groups for *en bloc* resection and complications after surgery, the curative resection rate was significantly lower in the discordant group (96.7% vs 86.8%, *P* = .002) But the results of the mean (SD) 37.82 (11.2) month follow-up period confirmed that there were no statistically significant differences for the long-term outcomes including local recurrence (*P* = .559) and overall mortality (*P* = 1.000). Both groups showed positive prognoses after endoscopic resection, which was attributed to the inclusion of only the absolute indication group for examination.

Endoscopic resections were performed because the patients were diagnosed with differentiated-type cases in the forceps biopsy, but there is still a lack of guidelines when histologic discrepancy is ultimately confirmed as an undifferentiated-type case. Because undifferentiated EGC is not included as an absolute indication for ESD, the standard regarding whether additional surgery or follow-ups are necessary, even when curative resections are performed, differs between doctors. As endoscopic resection techniques and methods develop and advance, the indication is expanding.^[3]^ Japanese gastric cancer treatment guidelines (2010, ver. 3) also regard ESD for these tumors as an investigational treatment for an investigational treatment that is smaller than 2 cm.^[19]^ Recent studies also report satisfactory therapeutic outcomes after performing ESD operations for undifferentiated EGC with small sizes when compared to that of the absolute indication group.^[20–23]^ While the curative resection rate was also confirmed to be lower in the discordant group when compared to that of the concordant group, there was no statistically significant difference in long-term clinical outcome, which is clinically significant. It can be concluded that the histologic discrepancy of EGC does not have an effect on the prognosis of patients undergoing endoscopic resection, and even if there is a diagnosis of the undifferentiated type in the final pathology results if curative resection has been conducted on the lesion, it is possible to follow-up and monitor these patients without additional treatment.

This study has limitations because of its retrospective design; therefore, there is a possibility of selection bias. However, it was determined that there was a small effect on the results owing to the large sample size of this study when compared to the results of previous research. Another limitation is the possibility of interobserver variation because no pathologist conducted the histological reviews. However, the histologic discrepancy rate of this study, which is the most important research factor, was similar to that of previous studies, and the variation was considered to be low because experienced GI pathologists performed the reviews. Previous research on histologic discrepancy was mainly limited to gastric adenoma, while there are few studies available on gastric cancer.^[9,14]^ This research is also strong in that it examined the long-term clinical outcomes of patients with the histologic discrepancy, an area that has not been researched before.

In conclusion, lesions located in the upper third of the stomach and whitish discoloration were risk factors for histological discrepancies between differentiated-type EGC and undifferentiated-type EGC. While the curative resection rate was low for groups in which histologic discrepancies occurred, there were no significant differences in the long-term prognosis of the patients. Therefore, if curative resections are performed on EGC patients with histologic discrepancies, it can be considered from the results of this research that follow-up on patients can be conducted without the need for additional treatment, but there is a need for additional research for verification.

## Acknowledgments

None.

## Author contributions

**Data curation:** Min-Kyung Yeo, Jae Ho Park.

**Investigation:** Min-Kyung Yeo, Sun Hyung Kang.

**Methodology:** Min-Kyung Yeo, Sun Hyung Kang.

**Writing – original draft:** Min-Kyung Yeo.

**Formal analysis:** Jae Ho Park.

**Conceptualization:** Sun Hyung Kang, Ju Seok Kim.

**Funding acquisition:** Hee Seok Moon, Ju Seok Kim.

**Project administration:** Hee Seok Moon, Hyun Yong Jeong.

**Resources:** Hee Seok Moon.

**Software:** Jae Kyu Sung.

**Validation:** Jae Kyu Sung, Ju Seok Kim.

**Writing – review & editing:** Jae Kyu Sung, Hyun Yong Jeong, Ju Seok Kim.

**Supervision:** Hyun Yong Jeong, Ju Seok Kim.
